# New potential biomarkers for early chronic kidney disease diagnosis in patients with different glucose tolerance status

**DOI:** 10.3389/fendo.2023.1206336

**Published:** 2023-07-06

**Authors:** Velia Cassano, Corrado Pelaia, Giuseppe Armentaro, Sofia Miceli, Valeria Tallarico, Daniele Dallimonti Perini, Vanessa T. Fiorentino, Egidio Imbalzano, Raffaele Maio, Elena Succurro, Marta L. Hribal, Francesco Andreozzi, Giorgio Sesti, Angela Sciacqua

**Affiliations:** ^1^ Department of Medical and Surgical Sciences, University Magna Græcia of Catanzaro, Catanzaro, Italy; ^2^ Department of Experimental and Clinical Medicine, University Magna Græcia of Catanzaro, Catanzaro, Italy; ^3^ Department of Clinical and Experimental Medicine, Polyclinic University of Messina, Messina, Italy; ^4^ Department of Clinical and Molecular Medicine, Sapienza University of Rome, Rome, Italy

**Keywords:** oxidative stress, renal function, glucose tolerance, endocan, platelets activation

## Abstract

**Background:**

The purpose of the present study was to investigate the role of oxidative stress, platelet activation, and endocan levels in renal dysfunction in normal glucose tolerance (NGT) patients with 1-h plasma glucose values ≥155 mg/dl (NGT ≥ 155), compared to NGT < 155, impaired glucose tolerance (IGT), and type 2 diabetes mellitus (T2DM) newly diagnosed subjects. We enlisted 233 patients subjected to an oral glucose tolerance test (OGTT).

**Materials and methods:**

The serum levels of platelet activation (glycoprotein VI and sP-selectin), oxidative stress biomarkers (8-isoprostane and Nox-2), and endocan were evaluated using an ELISA test.

**Results:**

Among NGT < 155 patients and the T2DM group, there was a statistically significant increase in 8-isoprostane (p < 0.0001), Nox-2 (p < 0.0001), glycoprotein VI (p < 0.0001), and sP-selectin (p < 0.0001) serum levels. Higher serum endocan levels were found with the worsening of metabolic profile (p < 0.0001); specifically, NGT ≥ 155 patients presented higher serum endocan values when compared to NGT < 155 patients (p < 0.0001). From the multivariate linear regression analysis, 1-h glucose resulted in the major predictor of estimated glomerular filtration rate (e-GFR) justifying 23.6% of its variation (p < 0.0001); 8-isoprostane and Nox-2 added respectively another 6.0% (p < 0.0001) and 3.2% (p = 0.001).

**Conclusion:**

Our study confirmed the link between 1-h post-load glucose ≥155 mg/dl during OGTT and the possible increased risk for chronic kidney disease (CKD) in newly diagnosed patients. The novelty is that we demonstrated a progressive increase in oxidative stress, platelet activation, and serum endocan levels with the worsening of metabolic profile, which becomes evident early during the progression of CKD.

## 1 Introduction

The progressive decline in renal function is a public health problem with a prevalence of >10% worldwide. Chronic kidney disease (CKD) determines patients with decreased renal function, independently from underlying pathophysiological processes (1). Patients with renal dysfunction, in particular subjects with advanced CKD, present an increased risk for cardiovascular (CV) events and dysfunction in the hemostatic system, in particular bleeding disorders and thrombosis (2).

In this context, oxidative stress has a central role in the pathophysiology, development, and complications of CKD; it is characterized by a disproportion between excessive oxidant radicals and inadequate degradation of radicals by antioxidant systems. Oxidant complexes such as reactive nitrogen species (RNS) and reactive oxygen species (ROS) are produced under physiological conditions and are eliminated by the antioxidant defense mechanism (3). In case of an imbalance in the equilibrium between prooxidants and antioxidants, oxidative stress leads to oxidative injury in cells, tissues, and organs (4). A deregulated equilibrium between ROS and reduction in the antioxidant system, which in turn increases oxidase enzyme activity and phagocyte activation and causes further oxidative stress, is a characteristic feature observed in CKD (4). The link between oxidative stress, CKD, and its complications is mediated by various mechanisms, such as increased nicotinamide adenine dinucleotide phosphate-oxidase (NADPH oxidases (NOX) activity), uremic toxin-induced endothelial nitric oxide synthase (eNOS), and decreased antioxidant defenses (5–7). Moreover, many studies reported that oxidative stress may alter the beneficial antioxidant properties of albumin, the most abundant circulating protein, which exerts important antioxidant activity and whose lower levels are associated with CKD progression (8, 9).

In addition, recent studies highlighted that endothelial dysfunction plays a significant role in CKD; in particular, endocan, a soluble proteoglycan secreted by endothelial cells and considered a novel biomarker of endothelial dysfunction, has been shown to contribute to various renal diseases such as diabetic nephropathy and autosomal dominant polycystic kidney disease (10). A study directed by Gunay et al. demonstrated that serum endocan levels were associated with endothelial dysfunction and inflammation in individuals with acute kidney injury (11); moreover, Cikrikcioglu and collaborators investigated the link existing between endocan levels and the early phase of diabetic nephropathy in patients with type 2 diabetes mellitus (T2DM), indicating that endocan may be a possible monitoring biomarker of the progression of diabetic nephropathy (12).

Subjects with 1-h plasma glucose values ≥155 mg/dl (normal glucose tolerance (NGT) ≥ 155), detected during oral glucose tolerance test (OGTT), are at increased risk to develop T2DM among subjects with normal glucose tolerance test (13). Moreover, recent observations highlighted that these subjects have a worse cardiometabolic risk profile and an increased risk for CKD (14–17). Recently, a study performed by our group proved that NGT ≥ 155 subjects exhibited prematurely increased levels of oxidative stress when compared to NGT < 155 subjects, indicating subclinical organ damage (18).

Based on past evidence present in the literature, the aim of the current study was to test the role of oxidative stress, platelet activation, and endocan levels in renal dysfunction in NGT < 155, NGT ≥ 155, impaired glucose tolerance (IGT), and T2DM individuals.

## 2 Materials and methods

### 2.1 Study population

We enlisted 233 Caucasian newly diagnosed hypertensive patients (132 men and 101 women, mean age 58.4 ± 11.0) referring to Catanzaro Metabolic Risk Factors (CATAMERI) Study (19). Exclusion criteria were causes of secondary hypertension, diagnosis of anemia, clinical evidence of heart failure, history of chronic or malignant respiratory disease, malabsorption diseases, endocrinological pathologies, alcohol, smoking, or drug abuse. No patients presented CKD. All participants underwent a review of their medical history, physical estimation, and anthropometrical evaluation with an evaluation of height, weight, and body mass index (BMI).

The ethics committee authorized the protocol, and informed written consent was acquired from all subjects (code protocol number 2012.63). All evaluations were performed in agreement with the principles of the Declaration of Helsinki.

### 2.2 Blood pressure measurement

Clinical blood pressure (BP) evaluations were acquired according to current guidelines. Measurements of BP were acquired in the left arm of patients in a sitting position, using a semi-automatic sphygmomanometer (OMRON, M7 Intelli IT) after 5 min of rest. BP values were the average of three measurements. This evaluation was repeated on three different occasions at least 2 weeks apart. Subjects with a clinic systolic BP (SBP) >140 mmHg and/or diastolic BP (DBP) >90 mmHg were defined as hypertensive (20). Pulse pressure (PP) values were acquired as the difference between systolic and diastolic BP measurements.

### 2.3 Laboratory determination

All laboratory determinations were executed after at least 12 h of fasting. A 75-g OGTT was executed with 0-, 30-, 60-, 90-, and 120-min sampling for insulin and plasma glucose. Glucose tolerance status was defined on the basis of OGTT using the World Health Organization (WHO) criteria, and T2DM was described according to the American Diabetes Association (ADA) criteria. Plasma glucose was estimated by the glucose oxidation method (Beckman Glucose Analyzer II; Beckman Instruments, Milan, Italy), and plasma insulin concentration was detected by a chemiluminescence-based assay (Roche Diagnostics, Milan, Italy).

Insulin sensitivity was estimated using the Matsuda index (insulin sensitivity index [ISI]), calculated as follows: 10.000/square root of [fasting glucose (millimoles per liter) × fasting insulin (milliunits per liter)] × [mean glucose/mean insulin during OGTT]. The Matsuda index is strongly related to the euglycemic–hyperinsulinemic clamp, which represents the gold standard test for measuring insulin sensitivity (21).

Serum creatinine was evaluated by an automated technique based on the Jaffè creatinine compensated method for plasma and serum (Roche Diagnostics) implemented in an auto-analyzer. Albumin concentration was estimated with an Alb2 kit on a Cobas C6000 analyzer (Roche Diagnostics, Milan, Italy). Triglycerides, total, high-density lipoprotein (HDL), and low-density lipoprotein (LDL) cholesterol values were determined by enzymatic methods (Roche Diagnostics, Mannheim, Germany). Values of estimated glomerular filtration rate (e-GFR) were determined by using the equation proposed in the chronic kidney disease epidemiology (CKD-EPI) collaboration (22).

#### 2.3.1 Serum levels of oxidative stress biomarkers, platelet activation, and endocan quantification

Blood samples, acquired from fasted subjects, were taken in tubes with separator gel and centrifuged for 15 min at 4,000 rpm to acquire serum samples that were directly stored at −80°C. In order to prevent oxidation, samples were stocked at −80°C in the presence of 0.005% butylated-hydroxy-toluene (BHT) (10 µl of 5 mg/ml solution in ethanol per 1-ml sample).

Quantitative determination of the serum 8-isoprostane (ELISA kit, Cayman Chemical, Ann Arbor, MI, USA) and Nox-2 (ELISA kit, MyBioSource, San Diego, CA, USA) was executed with commercial ELISA immunoassays in accordance with the manufacturer’s instructions. The 8-isoprostane values were reported as pg/ml; the lower detection limit of the assay was 0.8 pg/ml. Levels of Nox-2 were reported as nmol/L, and the lower detection limit of the assay was 0.25 nmol/L. The coefficient of variation (CV %) was <9%. Serum levels of human sP-selectin and glycoprotein VI were quantified using commercial ELISA immunoassays according to the manufacturer’s instructions (ELISA kit MyBioSource, San Diego, CA, USA). Values of glycoprotein VI were reported as pg/ml, the lowest detectable concentration was 46.88 pg/ml, and the CV (%) was <8%; values of sP-selectin were expressed in ng/ml, and the minimum detectable concentration was 15 ng/ml.

Quantitative determination of serum endocan levels was detected with a commercial ELISA immunosorbent assay kit (Abcam, Cambridge, MA< USA). Values of endocan were reported as ng/ml, the sensitivity was 0.12 ng/ml, and CV (%) was 4.2%.

### 2.4 Statistical methods

Normally distributed data were reported as mean ± SD. To investigate the differences between groups, analysis of variance (ANOVA) for biological and clinical was performed, followed by the Bonferroni *post-hoc* test for multiple comparisons. A chi-squared test was considered for categorical variables. A linear correlation analysis was performed in the entire study population, with the aim to evaluate the possible correlation between e-GFR, considered as dependent variables, and different covariates. Subsequently, variables achieving statistical significance were inserted in a multiple stepwise multivariate linear regression model to detect the independent predictor of e-GFR. Data were considered significant at p < 0.05. All comparisons were executed using the SPSS 20.0 for Windows (SPSS Inc., Chicago, IL, USA) statistical package.

## 3 Results

### 3.1 Study population

In the present study population, 233 patients were divided into four groups: 77 were NGT < 155, 57 were NGT ≥ 155, 61 were IGT, and 38 were T2DM. The mean age was 58.4 ± 11.0. The demographic, biochemical, and clinical characteristics of the patients are reported in **Table 1**, in accordance with distinct different metabolic states.

**Table 1:** Anthropometric, biochemical, and hemodynamic characteristics of the study population according to glucose tolerance status.

Variables	All (n = 233)	NGT < 155 (n = 77)	NGT ≥ 155 (n = 57)	IGT (n = 61)	T2DM (ND) (n = 38)	p^a^
Gender, M/F	132/101	37/40	30/27	40/21	25/13	0.113*
Age, years	61.4 ± 10.7	58.0 ± 12.2	65.0 ± 8.0	61.6 ± 11.2	62.2 ± 9.4	0.120
BMI, kg/m^2^	31.2 ± 6.5	30.3 ± 6.5	31.6 ± 6.2	32.2 ± 7.41	31.0 ± 4.8	0.402
SBP, mmHg	133.7 ± 8.6	130.8 ± 11.1	134.6 ± 6.2	135.4 ± 6.6	135.6 ± 7.4	0.031
DBP, mmHg	79.9 ± 8.1	77.8 ± 8.2	80.9 ± 8.6	80.9 ± 7.6	81.4 ± 7.4	0.766
PP, mmHg	54.7 ± 12.2	51.1 ± 13.1	53.4 ± 13.2	56.2 ± 11.1	60.4 ± 8.8	0.061
FPG, mg/dl	96.8 ± 12.3	87.6 ± 8.2	95.9 ± 8.9	100.4 ± 10.7	111.1 ± 9.7	<0.0001
1-h glucose, mg/dl	177.0 ± 45.5	128.9 ± 19.0	174.0 ± 23.5	200.2 ± 22.6	241.6 ± 24.1	<0.0001
2-h glucose, mg/dl	150.0 ± 46.1	112.0 ± 21.1	126.6 ± 11.5	174.7 ± 14.6	227.4 ± 23.5	<0.0001
Fasting insulin, μU/ml	15.2 ± 6.2	9.9 ± 2.7	15.0 ± 4.0	18.7 ± 6.4	20.8 ± 4.9	<0.0001
1-h insulin, μU/ml	101.8 ± 48.5	79.0 ± 47.7	119.6 ± 25.0	120.0 ± 60.2	92.2 ± 32.5	0.001
2-h insulin, μU/ml	105.6 ± 55.7	66.6 ± 27.6	95.0 ± 24.5	140.3 ± 58.6	144.6 ± 67.1	<0.0001
Matsuda/ISI	54.3 ± 28.5	84.8 ± 28.2	47.1 ± 10.4	36.9 ± 10.2	31.3 ± 8.7	<0.0001
Na, mmol/L	140.2 ± 2.6	140.3 ± 2.9	140.4 ± 2.8	139.8 ± 2.4	140.4 ± 1.6	0.480
K, mmol/L	4.2 ± 0.4	4.1 ± 0.3	4.3 ± 0.4	4.2 ± 0.4	4.2 ± 0.3	0.347
P, mg/dl	3.3 ± 0.5	3.3 ± 0.5	3.4 ± 0.5	3.2 ± 0.5	3.3 ± 0.5	0.393
Ca, mmol/L	9.3 ± 0.4	9.2 ± 0.4	9.3 ± 0.5	9.3 ± 0.4	9.3 ± 0.4	0.190
Total cholesterol, mg/dl	182.3 ± 33.1	185.6 ± 32.9	185.1 ± 27.3	175.7 ± 32.6	181.8 ± 41.3	0.280
Triglyceride, mg/dl	139.8 ± 63.6	135.2 ± 63.4	133.0 ± 45.7	139.2 ± 66.4	160.2 ± 66.4	<0.0001
HDL, mg/dl	48.3 ± 11.2	51.9 ± 10.8	49.7 ± 11.9	45.5 ± 9.6	43.3 ± 11.0	0.170
LDL, mg/dl	118.5 ± 31.0	120.7 ± 29.5	119.0 ± 28.8	114.7 ± 30.8	119.2 ± 37.4	0.576
hs-CRP, mg/L	3.1 ± 2.6	1.5 ± 1.0	3.4 ± 1.5	4.2 ± 3.7	4.4 ± 2.2	<0.0001
e-GFR, ml/min/1.73 m^2^	104.7 ± 18.0	119.2 ± 12.0	102.7 ± 13.8	95.1 ± 16.5	95.2 ± 17.0	<0.0001
Albumin, g/dl	4.5 ± 0.3	4.5 ± 0.3	4.5 ± 0.3	4.5 ± 0.3	4.3 ± 0.2	0.001
Albuminuria, mg/dl	16.5 ± 8.8	12.4 ± 6.5	16.3 ± 7.9	17.0 ± 8.2	24.2 ± 10.0	<0.0001
Creatinine, mg/dl	0.8 ± 0.1	0.7 ± 0.1	0.8 ± 0.1	0.8 ± 0.1	0.8 ± 0.1	<0.0001
Azotemia, mg/dl	35.3 ± 11.3	32.1 ± 10.0	34.9 ± 9.2	36.6 ± 10.3	40.4 ± 15.5	0.002
PLT, 10^3^/µl	215.2 ± 56.0	200.8 ± 46.3	214.2 ± 62.0	223.2 ± 58.2	232.6 ± 55.5	0.017
MPV, fl	9.1 ± 1.2	7.8 ± 0.6	8.4 ± 0.5	9.9 ± 0.6	11.2 ± 1.4	<0.0001

Data are mean ± SD.

NGT, normal glucose tolerance; IGT, impaired glucose tolerance; T2DM, type 2 diabetes mellitus; BMI, body mass index; SBP, systolic blood pressure; DBP, diastolic blood pressure; PP, pulse pressure; FPG, fasting plasma glucose; Na, sodium; K, potassium; P, phosphorus; Ca, calcium; HDL, high-density lipoprotein; LDL, low-density lipoprotein; hs-CRP, high-sensitivity C-reactive protein; e-GFR, estimated glomerular filtration rate; PLT, platelets; MPV, mean platelet volume.

a Overall difference among groups (ANOVA).

^*^ Overall differences among groups (χ^2^).

Among the four study groups, no significant differences were observed regarding age and gender distribution, anthropometric indicators, DBP, PP, total, LDL, and HDL cholesterol.

By contrast, among the study groups, there was a significant increase in SBP (p = 0.031), triglyceride (p < 0.0001), fasting plasma glucose (FPG) (p < 0.0001), 1-h glucose (p < 0.0001), and 2-h glucose (p < 0.0001) during OGTT, as well as fasting insulin (p < 0.0001), 1-h insulin (p < 0.0001), and 2-h insulin (p < 0.0001). As awaited, there was a worsening of insulin sensitivity accounting for the decrease of Matsuda/ISI (p < 0.0001). Moreover, we observed a worsening of the inflammatory profile with the deterioration of glucose tolerance, as attested by high-sensitivity C-reactive protein (hs-CRP) values (p < 0.0001) and an increase in platelet count (p = 0.017) and mean platelet volume (MPV) (p < 0.0001).

*Post-hoc* analysis by Bonferroni test proved that NGT ≥ 155 patients exhibited significantly reduced insulin sensitivity as showed by lower Matsuda/ISI (47.1 ± 10.4 *vs.* 84.8 ± 28.2, p < 0.0001) and higher values of hs-CRP (3.4 ± 1.5 *vs.* 1.5 ± 1.0, p < 0.0001) when compared with NGT < 155 patients and similar to the IGT group. In addition, NGT ≥ 155 subjects presented higher MPV values in comparison with NGT < 155 subjects (p < 0.0001).

As already demonstrated, there was a decrease in renal function, as demonstrated by e-GFR values, with the deterioration of metabolic status (p < 0.0001); moreover, there was a statistically significant rise in creatinine (p < 0.0001) and azotemia values (p = 0.002). Comparison between groups highlighted a worsening of renal function in NGT ≥ 155 subjects, as evidenced by decreased values of e-GFR (102.7 ± 13.8 *vs.* 119.2 ± 12.0, p < 0.0001) when compared to NGT < 155 subjects.

Moreover, serum albumin levels were significantly decreased with the worsening of glucose tolerance status (p = 0.001).

Among NGT < 155 patients and the T2DM group, there was a statistically significant increase in oxidative stress parameters such as 8-isoprostane (p < 0.0001) and Nox-2 (p < 0.0001) serum levels, denoting a rise in oxidative stress levels, with the worsening of metabolic profile. Particularly, NGT ≥ 155 subjects presented increased values of 8-isoprostane (43.6 ± 9.7 *vs.* 30.1 ± 5.5, p < 0.0001) and Nox-2 (1.2 ± 0.2 *vs.* 0.9 ± 0.1, p < 0.0001) when compared to NGT < 155 and comparable to IGT patients. Furthermore, among groups, a statistically significant increase was observed in platelet activation parameters such as glycoprotein VI (p < 0.0001) and sP-selectin (p < 0.0001).

A comparison between groups demonstrated that NGT ≥ 155 subjects had increased values of glycoprotein VI (60.2 ± 14.2 *vs.* 47.3 ± 4.6, p < 0.0001) and sP-selectin (109.9 ± 37.7 *vs.* 84.9 ± 20.6, p < 0.0001) in comparison with NGT < 155 subjects.

**Figure 1:**
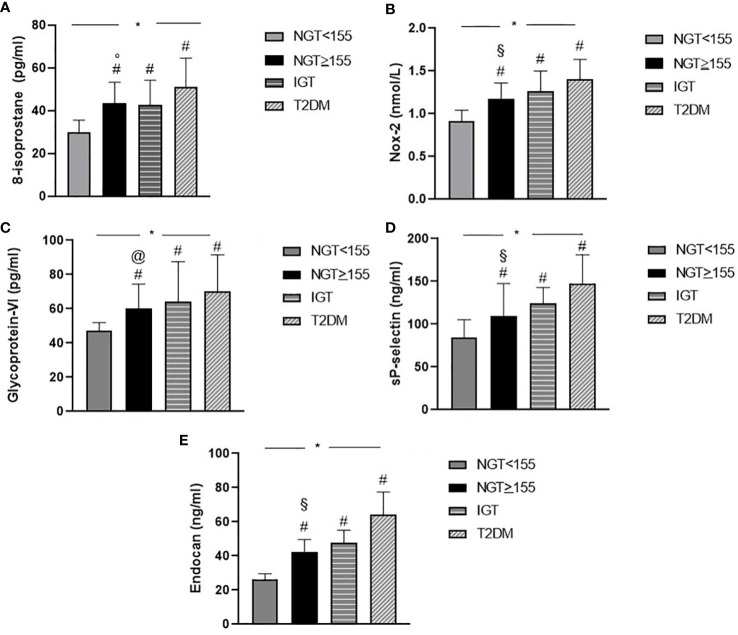
Graphical illustration of serum biomarkers levels of oxidative stress 8-isoprostane **(A)**, Nox-2 **(B)**, platelet activation glycoprotein VI **(C)**, sP-selectin **(D)**, and serum endocan **(E)** levels of the study population according to glucose tolerance status. *p < 0.0001, overall difference among groups (ANOVA). # p < 0.0001 other groups (NGT < 155, IGT, and T2DM) *vs.* NGT < 155 (Bonferroni *post-hoc* test). § p < 0.0001 NGT ≥ 155 *vs.* T2DM (Bonferroni *post-hoc* test). ° p = 0.001 NGT ≥ 155 *vs.* T2DM (Bonferroni *post-hoc* test). @ p = 0.030 NGT ≥ 155 *vs.* T2DM (Bonferroni *post-hoc* test). NGT, normal glucose tolerance; IGT, impaired glucose tolerance; T2DM, type 2 diabetes mellitus.

Of interest, higher serum endocan levels were found with the worsening of metabolic profile (p < 0.0001); in particular, NGT ≥ 155 patients presented higher serum endocan values when compared to NGT < 155 patients (42.4 ± 7.2 *vs.* 26.1 ± 3.4, p < 0.0001) (**Figure 1**).

### 3.2 Correlation analysis

From the linear correlation analysis, e-GFR resulted significantly and negatively correlated with 1-h glucose (r = −0.489, p < 0.0001), 8-isoprostane (r = −0.473, p < 0.0001), Nox-2 (r = −0.479, p < 0.0001), endocan (r = −0.476 p < 0.0001), glycoprotein VI (r = −0.238, p < 0.0001), sP-selectin (r = −0.368, p < 0.0001), and hs-CRP (r = −0.171, p = 0.005) and positively correlated with Matsuda/ISI (r = 0.418, p < 0.0001) (**Table 2**).

**Table 2:** Linear correlation analysis between e-GFR, as dependent variables, and different covariates in the entire study population.

	e-GFR
	r/p
Matsuda/ISI	0.418/<0.0001
1-h glucose, mg/dl	−0.489/<0.0001
8-isoprostane, pg/ml	−0.473/<0.0001
Nox-2, nmol/L	−0.479/<0.0001
Endocan, ng/ml	−0.476/<0.0001
Glycoprotein VI, pg/ml	−0.238/<0.0001
sP-selectin, ng/ml	−0.368/<0.0001
h-CRP, mg/L	−0.171/0.005

e-GFR, estimated glomerular filtration rate; hs-CRP, high-sensitivity C-reactive protein.

Variables reaching statistical significance were included in a stepwise multivariate linear regression model to detect the independent predictors of e-GFR; 1-h glucose resulted in the major predictor of e-GFR justifying 23.6% of its variation (p < 0.0001); 8-isoprostane and Nox-2 added respectively another 6.0% (p < 0.0001) and 3.2% (p = 0.001) (**Table 3**).

**Table 3:** Stepwise multiple regression analysis on e-GFR in the entire study population.

	e-GFR		
	Partial R^2^	Total R^2^	p
1-h glucose, mg/dl	23.6	23.6	<0.0001
8-isoprostane, pg/ml	6.0	29.6	<0.0001
Nox-2, nmol/L	3.2	32.8	0.001

e-GFR, estimated glomerular filtration rate.

## 4 Discussion

In the current study, we investigated the association among oxidative stress biomarkers, platelet activation, endocan serum levels, and renal function in newly diagnosed hypertensive patients subjected to OGTT, highlighting also that hypertension is a risk factor for CKD disease. The patients were divided into four groups (NGT < 155, NGT ≥ 155, IGT, and T2DM) according to glucose tolerance status and considering the cutoff point of ≥155 mg/dl at 1 h during OGTT. Serum levels of endocan and biomarkers of oxidative stress and platelet activation were significantly higher in NGT ≥ 155 subjects than in NGT < 155 subjects and comparable to those in IGT subjects. Moreover, data acquired from the current study confirmed that NGT ≥ 155 patients not only are at increased risk to develop T2DM and CV disease but are also at increased risk to develop CKD. In detail, NGT ≥ 155 patients presented lower values of e-GFR, the most commonly employed marker of kidney function, than did NGT < 155 patients. It is interesting that in a linear correlation analysis, e-GFR was significantly correlated with oxidative stress, platelet activation, serum endocan, and hs-CRP; moreover, from the stepwise multivariate linear regression model, 1-h glucose resulted as the major predictor of the worsening of e-GFR, justifying 23.6% of its variation, and 8-isoprostane and Nox-2 were responsible respectively for 6.0% and 3.2% of the e-GFR variation. In addition, there was a progressive reduction in albumin, from the first to the fourth group, although it remained in the range of reference values, attributable to the increase in oxidative stress (8). However, it is important to highlight that despite high blood pressure values and high glucose levels, none of the patients had CKD, and creatinine and e-GFR values were within the normal range values.

In spite of their well-established relationship, complex interactions between oxidative stress, endothelial dysfunction, and renal damage make it arduous to differentiate which process is principally responsible for initiating the series of events that possibly lead to kidney failure.

Mechanistically, chronic hyperglycemia increases oxidative stress, and oxidative stress, endothelial dysfunction, and inflammation are detectable even at a very early stage of CKD (23, 24). The principally pathological mechanism that correlates oxidative stress with CKD progression is defined by an early impairment in the kidney due to the activities of intra- and extracellular oxygen-derived radicals and the resultant inflammatory response. The free radical molecules ROS interact with molecular components of nephrons, resulting in decreased membrane viability and cleavage and cross-linking of renal DNA with the consequence of malignant mutations and immediate nephron damage (25). Numerous intracellular mechanisms are implicated in ROS production at the renal level, involving cytochrome P450 system, xanthine oxidase, mitochondrial respiratory chain, and NOXs. Of interest, NOXs are particularly relevant since they have been recognized to generate ROS not as by-products but as their sole biological function (26). Under physiological conditions, NOXs present very low or no constitutive activity, but their expression may be increased in pathological states such as hypertension and diabetes. In experimental *in vitro* models, NOX isoforms are upregulated in glomerular cells in response to high glucose levels (27). The Nox-2 isoform, the classic prototype of NOX isoforms expressed in mesangial cells, endothelial cells, vascular smooth muscle cells, immune cells, tubular epithelial cells, and podocytes, contributes to renal injury. Data acquired from our study demonstrated that there was a significant increase in serum Nox-2 levels, from the first to the fourth group, denoting an increase of oxidative stress with the deterioration of metabolic status, and the linear correlation analysis highlighted the link between oxidative stress, inflammation, and renal function. In addition, in our study, we observed higher serum 8-isoprostane levels and decreased e-GFR with the deterioration of glucose tolerance status. In fact, the 8-isoprostane, an accurate marker of endogenous lipid peroxidation and oxidative stress, increases prematurely during CKD progression (28). Moreover, low serum albumin levels may rely on several mechanisms involving inflammation, nutritional issues, and renal disease (29). Antecedent studies described that, in T2DM patients, albumin undergoes glycation, loses its antioxidant properties, and may exhibit prooxidant properties. These modifications may have an adverse influence on the coagulation system, as albumin has antiplatelet and anticoagulant characteristics through mechanisms related to its antioxidant effects; in particular, albumin impairs platelet aggregation with a mechanism connected to Nox-2 downregulation.

In this context, it is important to emphasize that renal dysfunction is associated with increasing molecule adhesion, a factor associated with platelet hyperactivity; furthermore, platelet activation in diabetic, obese, and hypertensive patients is imputed to endothelial dysfunction, oxidative stress, subclinical inflammation, and altered renin–angiotensin aldosterone system (RAAS) (2, 30). To investigate this issue, we analyzed platelet activation biomarkers in the complete study population and the four subgroups in accordance with glucose tolerance status. We observed a progressive increase of sP-selectin and glycoprotein VI serum levels, with the worsening of glucose tolerance status and the progressive decrease of e-GFR.

In detail, platelets’ surface can present higher levels of p-selectin and glycoprotein VI, denoting a release of α-granules. Furthermore, p-selectin promotes the formation of platelet–erythrocyte aggregates in subjects with advanced CKD (2). Platelet erythrocyte aggregation and platelet hyperactivation are involved in the increased risk of thrombosis. These molecular mechanisms may explain why CKD patients present an increased risk of thrombotic and CV complications.

Oxidative stress has also a main role in progressive endothelial dysfunction, which has a main role in promoting atherogenesis in CKD subjects. Endocan, a marker involved in many pathological conditions such as endothelial dysfunction and inflammation, is increased in CKD patients. Results obtained from our study demonstrated a progressive increase of endocan serum levels with the worsening of metabolic status, and a strong and inverse correlation between endocan and e-GFR; however, endocan did not enter into stepwise multivariate linear regression model because of collinearity with oxidative stress biomarkers. A previous study conducted by Yilmaz et al. proved that plasma endocan was correlated with e-GFR; in fact, subjects with CKD presented higher endocan levels compared to control subjects (31).

In conclusion, our study confirmed the link between 1-h post-load glucose ≥155 mg/dl during OGTT and the possible increased risk for CKD in newly diagnosed patients. The novelty of the present study is that we demonstrated a progressive increase in oxidative stress and platelet activation with the worsening of metabolic profile and significantly early during the progression of CKD; in particular, NGT ≥ 155 patients exhibited higher serum levels when compared to NGT < 155 patients. Moreover, our data demonstrated a progressive increase in serum endocan levels, an emerging molecule secreted by endothelial cells, and it seems to be involved in many pathological processes such as CKD. Furthermore, more studies are needed to investigate whether endocan is only a marker of a negative prognosis in these conditions or has an active role. In addition, biomarkers evaluated in this study could be used as potential biomarkers in early diagnosis of CKD in patients with different glucose tolerance status.

However, the present study has a study limitation; in fact, the markers analyzed in this study underlie the molecular mechanisms of interconnected diseases, in particular the high levels of oxidative stress caused by chronic hyperglycemia, which have a negative effect on the systemic level. Therefore, the values of these biomarkers should be contextualized inherently to the patient’s clinical phenotype.

## Data availability statement

The raw data supporting the conclusions of this article will be made available by the authors, without undue reservation.

## Ethics statement

The studies involving human participants were reviewed and approved by Comitato Etico Azienda Ospedaliera “Mater Domini”. The patients/participants provided their written informed consent to participate in this study. The ethics committee approved the protocol, and informed written consent was obtained from all participants (code protocol number 2012.63). All investigations were performed in accordance with the principles of the Declaration of Helsinki.

## Author contributions

Conceptualization: AS and VC Methodology: VC. Formal analysis: VC and CP. Investigation: VC and SM. Writing—original draft preparation: VC and CP. Writing—review and editing: AS, MLH, and GA. Supervision: AS, MLH, RM, GA, VT, DDP, ES, FA, GS, VTF, and EI. All authors contributed to the article and approved the submitted version.
